# Endothelial Cells as Tools to Model Tissue Microenvironment in Hypoxia-Dependent Pathologies

**DOI:** 10.3390/ijms22020520

**Published:** 2021-01-07

**Authors:** Aleksandra Majewska, Kinga Wilkus, Klaudia Brodaczewska, Claudine Kieda

**Affiliations:** 1 Laboratory of Molecular Oncology and Innovative Therapies, Military Institute of Medicine, PL-04-141 Warsaw, Poland; kwilkus@wim.mil.pl (K.W.); kbrodaczewska@wim.mil.pl (K.B.); ckieda@wim.mil.pl (C.K.); 2 Postgraduate School of Molecular Medicine, Medical University of Warsaw, PL-02-091 Warsaw, Poland; 3 Center for Molecular Biophysics UPR 4301 CNRS, 45071 Orleans, France

**Keywords:** alternative methods, angiogenesis, endothelial cells, endothelial progenitors, hypoxia, induced pluripotent stem cells, in vitro 3-dimensional models, microenvironment, organo-specificity

## Abstract

Endothelial cells (ECs) lining the blood vessels are important players in many biological phenomena but are crucial in hypoxia-dependent diseases where their deregulation contributes to pathology. On the other hand, processes mediated by ECs, such as angiogenesis, vessel permeability, interactions with cells and factors circulating in the blood, maintain homeostasis of the organism. Understanding the diversity and heterogeneity of ECs in different tissues and during various biological processes is crucial in biomedical research to properly develop our knowledge on many diseases, including cancer. Here, we review the most important aspects related to ECs’ heterogeneity and list the available in vitro tools to study different angiogenesis-related pathologies. We focus on the relationship between functions of ECs and their organo-specificity but also point to how the microenvironment, mainly hypoxia, shapes their activity. We believe that taking into account the specific features of ECs that are relevant to the object of the study (organ or disease state), especially in a simplified in vitro setting, is important to truly depict the biology of endothelium and its consequences. This is possible in many instances with the use of proper in vitro tools as alternative methods to animal testing.

## 1. Introduction

A monolayer of endothelial cells (ECs composing the endothelium) lines the entire vascular system—blood and lymphatic vessels—forming an interface between circulating fluids and vessel wall. Endothelium plays a critical role in maintaining the homeostasis of the body through: regulation of blood pressure, blood coagulation and fibrinolysis, achieving the active transport of molecules and the participation in immunological processes (adhesion and/or transmigration of inflammatory cells and specific homing of immune cells) which is documented in many examples [1].

**Figure 1:**
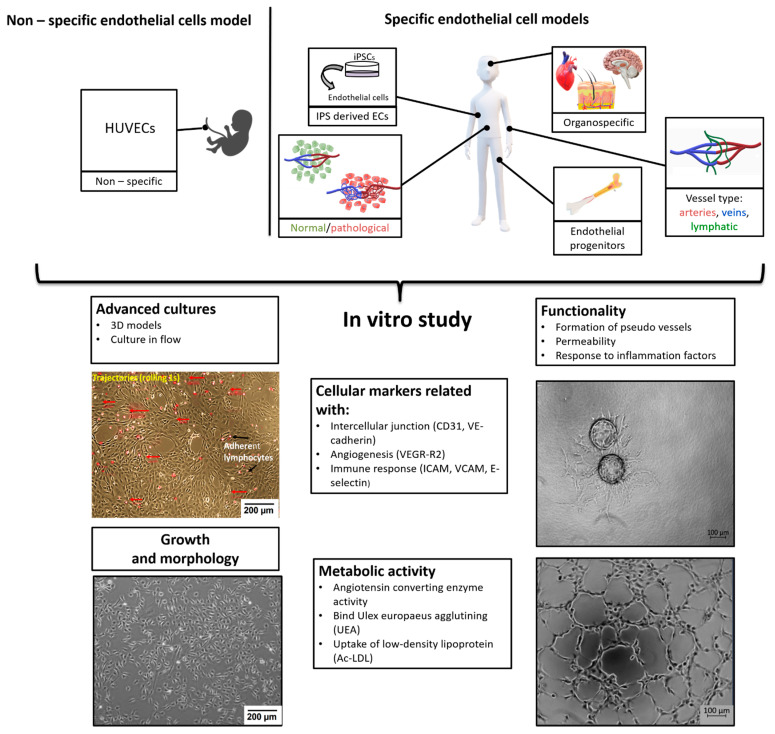
Organospecificity and plasticity of endothelial cells, selective angiogenesis model for signaling in pathologies and repair.

Due to the widespread occurrence of the vascular system in the body—endothelial cells are pervasive and occur in all parts of the organism—the weight of endothelium in an adult human is about 720 g [2]. However, endothelium is not a set of identical cells, but it is an extremely phenotypically diverse system (Figure 1). Another feature is structural, ECs differ according to their origin i.e., whether they are from microvessels (FSkMEC), macrovessels (FUmEC) (Patent number: 9228173) or lymphatics vessels (SVHEC SV40 immortalized murine endothelium cells line from peripheral lymph-node high endothelium) [3,4]. Moreover, activity of ECs varies in pathological states as compared to physiological ones (healthy breast-derived ECs and breast tumor-derived ECs). Differences are visible at the level of gene expression, surface antigens, cell morphology and properties linked to the biological state of the organ they are in. In this summary, we present various endothelial cell models used in in vitro studies, taking into account distinct states of cell differentiation, organo-specificity, origin and functions, what will be valuable for the selection of proper, relevant to in vivo conditions, research model. The selection of ECs as models for in vitro studies requires detailed characteristics.

This review will consequently present a synthesis of the advancements of the knowledge about endothelial cells properties and show their necessity to design proper methods for the study of diseases. Indeed, a huge progress in deciphering the molecular mechanisms of pathologies progression is being achieved, pointing to the role of hypoxia. The lack of access to oxygen makes it crucial in the relationship of diseased cells to the vasculature and its state as deficient vs normal. Consequently, the angiogenesis-related strategies are very actively studied. They necessitate the design of relevant models that require a strong knowledge of the endothelium characteristics and biological properties.

## 2. Characteristic and Functions of EC

### 2.1. The Phenotype of ECs

The development of endothelial cells (ECs) from the mesoderm begins in the early stages of embryo gastrulation. During the process of vasculogenesis, endothelial progenitor cells (angioblasts) form a de novo primitive vascular plexus, which later differentiates into arterial, venous, lymphatic and capillary EC [5,6]. This process is also observed in adults during the recruitment of bone marrow progenitors in response to ischemic injury [7].

Endothelial cells are characterized on many levels (Figure 1): cell growth and morphology, cellular markers, metabolism and functionality. In standard 2D (two-dimensional) cultures, endothelial cells have cobblestone shape but in more advanced models with dynamic flow, due to shear stress, the cells elongate and more closely mimicking the shape, and thus the physiology, of the vessel in vivo [1]. Due to the high heterogeneity of endothelial cells in terms of surface and cytoplasmic markers, we can distinguish: markers universal for ECs such as CD31 (PECAM-1 Platelet endothelial cell adhesion molecule) [8] or VE-cadherin (CD144) [9], CD133 for endothelial progenitors [10] or function specific vessel markers such as Claudin-5 for tight junction in cerebral and lung ECs [11]. There are also markers specific to vessel types: LYVE-1 (Lymphatic vessel endothelial hyaluronan (HA) receptor-1) or in some cases IL-7 receptor for lymphatic endothelial cell [12], Ephrin-B2 for arterial endothelial cells, whereas EphB4 (Ephrin type-B receptor 4) marks venous endothelial cells [13]. Ephrin and Eph receptor tyrosine kinase families play an important role in angiogenesis and vasculogenesis both in development as in pathological processes [14]. ErphinA1 expression is strongly upregulated in hypoxic conditions in cancer cells and promotes angiogenesis through a coordinated cross-talk with PI3K/AKT dependent endothelial nitric oxide synthase (eNOS) activation [15]. The most commonly used endothelial cell markers are summarized in the Table 1.

The assessment of typical endothelial biochemical pathways is another parameter of the endothelial cell characterization. Endothelial cells exhibit angiotensin converting enzyme activity (ACE; CD143), involved in the metabolism of angiotensin and inactivation of bradykinin [26]. ECs express receptors for acetylated low-density lipoprotein (Ac-LDL), which can be easily detected after incubation with labeled ligand (DiL-Ac-LDL) [27]. Binding of Ulex europaeus agglutining (UEA) is also characteristic for endothelial cells [28].

### 2.2. Regulation of ECs’ Heterogeneity

As Aird W.C. describes, endothelial cell heterogeneity is associated with epigenetic modifications (DNA methylation, histone methylation and histone acetylation), caused by extracellular signals, which negatively or positively affect gene expression. Epigenetic changes can persist after removal of signals and are transmitted during mitosis. It is different in the case of the micro-environmental impact, which also influences endothelial cells’ heterogeneity—it is represented by receptor-mediated posttranslational modification of transcription factors and other proteins, but the removal of the external factor causes a loss of such effects and a phenotype change linked to each subsequent mitosis [29]. These changes make the primary endothelial cells ineffective in in vitro studies because their phenotype becomes less specific with each passage due to the lack of a unique microenvironment. Chi et al. demonstrated that ECs isolated from different sites of the human vasculature after multiple passages had different transcriptional profiles between not only macrovascular and microvascular ECs but also between arterial and venous ECs [30]. These findings were supported by Lacorre et al. who showed that in vitro culture of differentiated ECs resulted in downregulation of genes, which were upregulated in natural tissue microenvironment [31]. These data indicate that epigenetics and microenvironment play role in mediating regulation of tissue-specific EC genes expression. Moreover, Burridge and Friedman, presented in their DNA microarray study, that origin influenced on differences in endothelial transcriptome of coronary and iliac arteries [32]. Taken altogether, heterogeneity of ECs may be caused by effects of different extracellular environments and epigenetic modifications induced by extracellular signals, and these have to be taken into account when culturing ECs in vitro, devoid of these signals. Not only epigenetic modification but also biochemical modification, such as glycosylation of the cells, affects modulation and regulation of biological processes [33,34].

### 2.3. microRNA Mediated ECs’ Modulation

Small non-coding RNA molecules (microRNA, miRNA) that regulate gene expression are also involved in maintaining homeostasis and ECs’ functionality. Fish et al. found that miR-126, which is known as an endothelial-specific miRNA, regulated the response of ECs to VEGF (vascular endothelial growth factor) by repressing negative regulators of the VEGF pathway, including the Sprouty-related protein (SPRED1) and phosphoinositol-3 kinase regulatory subunit 2 (PIK3R2/p85-β) what promoted angiogenesis and indicated that miR-126 regulates vascular integrity [35]. miR-31 and miR-17-3p induced by TNFα (tumor necrosis factor α) are associated with increased expression of adhesion molecules in ECs (selectin-E and ICAM-1), therefore are involved in maintaining important immunological functions of ECs [36]. In in vitro, studies miR-125b was shown to inhibit the translation of VE-Cadherin mRNA and tube formation by ECs, in-vivo miR-125b induced nonfunctional blood vessel formation [37]. Regulation of angiogenesis at the miRNA level (both promoting and inhibiting) is important in many pathological states. Proagniogenic miRNAs include, for example, miR494 targeting PTEN (phosphatase and tensin homolog deleted on chromosome ten) in lung cancer [38] or miR -155 targeting VHL (Von Hippel–Lindau tumor suppressor) in breast cancer [39], in turn, miR622 has an anti-angiogenic effect through by suppressing the CXCR4-VEGFA axis in colorectal cancer [40]. The effect of the microRNA may differ depending on the type of tumor—miR-27-b has pro-angiogenic activity in lung [41] but anti-angiogenic in ovarian cancer [42]. MicroRNA-103 regulates the vascular permeability by inhibiting VE-cadherin as well as other molecules associated with endothelial integrity as p120-catenin (p120) and zonula occludens 1 (ZO-1) which results in the promotion of metastasis [43]. Patients with ischemic stroke had a low miR-221 level and a study by Peng H. et al. confirmed that miR-221 caused a decrease in the viability and migration of ECs by targeting the PTEN/PI3K/AKT pathway [44]. The above examples show that miRNAs, originating from ECs and surrounding cells, regulate ECs’ activity and functionality by targeting various signaling pathways and modulate EC function on many levels. This regulation plays an important role in many pathological states, which shows the possibilities of using miRNA-based therapies acting on endothelial cells. The detailed role of miRNAs in endothelial cells has been reviewed elsewhere [45,46,47].

### 2.4. Functional Heterogeneity of ECs

**Figure 2:**
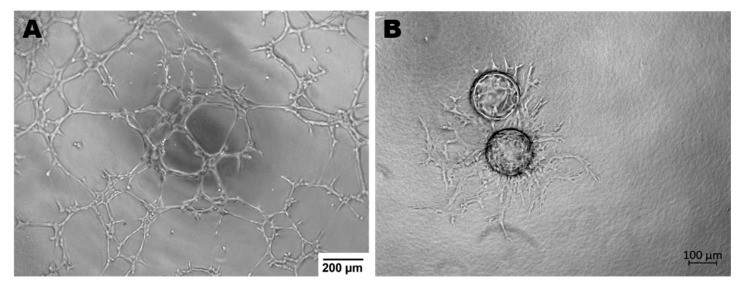
Endothelial cells ability to angiogenesis in vitro. (**A**)—Tube Formation Assay, magnification 4×; (**B**)—Sprouting Assay based on Cytodex beads, magnification 5×; both assays were performed with murine brain derived endothelial cells (MBr MEC FVB).

Endothelial cells display many functions, most of which are performed by vascular beds or specific subsets of blood vessel types. Apart from hemostasis, leukocyte trafficking or regulation of vasomotor tone, ECs take part in angiogenesis, permeability and acquired immunity [48]. Angiogenesis is the process of forming vessels from pre-existing ones and plays an important role in physiological processes (e.g., in adults: wound healing, in the female reproductive tract under control of the estrous cycle). On the other hand, pathological angiogenesis is associated with the development of many diseases such as cancer, rheumatoid arthritis or retinopathies [49]. The tube formation assay or sprouting assay are used to test the functionality of ECs in in vitro conditions (Figure 2) [50,51].

In addition to testing for angiogenesis, further functional tests based on EC properties include the endothelial permeability assay which is particularly important in studies related to the blood–brain barrier or metastasis. Vascular permeability is closely related to the TEER (Transepithelial/transendothelial electrical resistance) value, which is a strong indicator of the integrity of the endothelial cell layer and is evaluated prior to drug and chemical transport testing [52]. In permeability tests, Transwell filters covered with ECs are usually used and after adding the test substance to the top of the filter, its concentration in the bottom of the filter is measured. Permeability is also tested in advanced microflow models, which further ensures shear stress, also affects permeability and better reflects in vivo conditions [53,54].

The function of endothelial cells is also associated with immunity and response to inflammation, by regulating the migration of immune cells through the vascular wall to inflammatory sites. Adhesion molecules (such as ICAM-1, VCAM-1, Selectins, PECAM-1) play an important role in this process [55]. Therefore, in in vitro conditions the degree of activation of adhesion molecules after cytokine stimulation can be assessed, such as E-Selectin expression after TNFα or IL-1 stimulation [16]. Functional studies include a simple test for the adhesion of immune cells to a monolayer of endothelial cells [56,57] or a more advanced microfluid test, where the rolling and adhesion of leukocytes can be observed in real time [20,58]. Bielawska-Pohl et al. showed that interaction between immune cells and organospecific endothelial cells can be a potential target to block vascular injury [59].

## 3. ECs for in vitro Research

### 3.1. Immature Endothelial Cells—Endothelial Progenitor Cells/Endothelial Precursor Cells

Endothelial progenitor cells (EPCs) are defined as cells that are able to differentiate into mature endothelial cells and contribute to the formation of new blood vessels. Putative EPCs were first described in 1997. Asahara T. et al. isolated cells enriched for expression of CD34 and Flk-1 (receptor for vascular endothelial growth factors A and B; VEGFR2) among peripheral blood mononuclear cells (MNCs) in the circulation. These putative EPCs, upon transplantation, localized in the vessels and contributed to promoting vascular regeneration at sites of ischemia [60]. This initiated an increased interest in the topic of EPC and the development of methods to use EPC to repair ischemic organ functions through enhanced vasculogenesis [61,62]. At the same time, many controversies related to the classification, characteristics and nomenclature of these cells arose. The main problems are: the lack of specific markers, as well as high heterogeneity of EPCs, associated with various proliferative and differentiation abilities and EC plasticity conditioned by the environmental composition which results in the lack of unequivocal data [34]. Expression of markers changes during the trafficking and during the process in which EPCs differentiate into endothelial cells—in bone marrow these cells express CD34, CD133 and VEGFR2, while in the circulation, they express CD31 and CD146, and once reaching maturity markers characteristic for ECs as VE-cadherin, von Willebrand factor (VWF) are detected [63]. EPCs types distinguished by their origin and isolation methods (MNCs culture or flow cytometry) were reviewed by Medina R.J. et al. [64]. The source of EPCs for in vitro tests is most often peripheral blood [60] and bone marrow [65], cord blood [66,67], but also fetal liver [68], aorta gonad [69] and adipose tissue [70]. As indicated by Yoder M.C. et al. various methods of EPC isolation provide cells with different phenotypes—capable or unable to integrate into existing vessels in human model [71].

Significant differences in both gene expression and functional abilities, were observed in immortalized endothelial cell progenitors isolated from mouse aorta-gonads: 10.5 or 11.5 days post conception (dpc)—MAgEC 10.5 and MAgEC 11.5, which represent a model of EPCs at different stages of maturation. Both lines highly expressed stem cell markers (Sca-1 and CD34), but also EPCs/mature endothelial cell markers (PECAM-1 (CD31)) and the angiotensin converting enzyme, VWF, while expression of EphB4 indicates their venous vessel commitment. Loss of CD133, increase of VE-cadherin, VEGFR2 and PODXL (podocalyxin-like protein 1) (phenotype associated with mature EC) protein expression were observed in more differentiated MAgEC11.5. There are also significant differences in the ability to form pseudo-vessels between these two cell types. MAgEC 11.5 generate pseudo-vascular structures and networks, while MAgEC 10.5 do not have such abilities, but acquire them after stimulation with CX3CL1 and CCL21, which confirms that MAgEC 10.5 is present in the phenotype of early progenitors. Both cell lines effectively cooperate with mature MLuMEC lung microvascular cells to produce pseudo vessels in vitro, which gives hope for their influence in neovascularization in diseases such as cancer [69,72].

Primary cells of EPC characteristics could be obtained by culture of MNCs isolated from human umbilical cord blood (HUCB)—up to day 3 of culture, cells display monocytic characteristics (CD14+, CD45+), but after 6 days in culture the expression pattern shifts to an endothelial character (cells positive for VEGFR2, VE-cadherin, took up Ac-LDL and bound the endothelial specific lectin UEA-1); however, CD133 and CD34 are not expressed. In the same study, the phenotype of EPCs obtained after prior selection of CD45+ cells was also assessed—these cells also expressed endothelial cells phenotype over time, but also retained progenitor markers CD133 [73].

Circulating (recruited from the bone marrow) endothelial cell progenitors appear to be an important marker and significance for potential therapy in many diseases, such as cancer [74,75] cardiovascular [76,77] or pulmonary diseases [78], endometriosis [79] and diabetes [80,81].

Two cell lines, human endothelial progenitor cells—cord blood: HEPC-CB.1 and HEPC-CB.2 were isolated from umbilical vein and immortalized in vitro as described the authors Paprocka et al. [67]. These cell lines are early progenitor cells and are characterized by high proliferation ability. Both HEPC-CB.1 and HEPC-CB.2 were investigated by flow cytometry in order to assess their immunophenotype. It has been shown, that they are positive for the following markers: general stem cells (CD133), hematopoietic stem cells (CD13), non-hematopoietic stem cells (CD271), mesenchymal stem cells (CD90) and endothelial cell markers; mature as CD202b, VEGFR2, CD146 and stem-like or activated as CD105 [67,82]. Both cell lines are positive for CXCR4 (fusin, CD184); the unique receptor for the CXCL12 (SDF1 stromal cell-derived factor 1) chemokine, and weakly express markers of stem cells and endothelial cells: CD44 and CD15s. Both, HEPC-CB.1 and HEPC-CB.2 were weakly positive for the markers of progenitors but also differentiated endothelial cells (UEA-1 and Dil-Ac-LDL). On the other hand, both cell lines did not present markers of hematopoietic origin (CD34, CD117, CD45) and were negative for some markers of differentiated endothelial cells (CD31 and VWF). Moreover, when cultured in various oxygen partial pressures, these lines showed different patterns of chemokine secretion. The levels of IL-8, VEGF and angiogenin were higher in hypoxia than in normoxia. Moreover, both cell lines secrete IL-6 and IL-10 (anti-angiogenic factor) [67]. Furthermore, they were CD133+, CD34−, VEGFR2+ and CD31− what characterizes immature endothelial progenitor cells [83]. In addition to the above, endothelial nitric oxide synthase (eNOS) mRNA was present in both cell lines confirming the endothelial character of these cells [67,84]. However, it should be mentioned that there were differences between both cell lines. Firstly, the higher expression of CD133 and lower of CD271 was presented by HEPC-CB.2 as compared to HEPC-CB.1 [82]. IL-10 was secreted at a lower level by HEPC-CB.2 than second cell line [67]. Taking into account migration ability, both cell lines were more prompt to migrate to the increasing VEGF-concentration medium. Although both cell lines present a clear angiogenic potential as indicated by the expression of receptors: VEGFR2, and CXCR4 the cells did not achieve angiogenesis in in vitro assay [67]. Taken altogether, both cell lines were positive for the early endothelial markers but did not or poorly express markers of differentiated endothelial cells and can provide a relevant in vitro model of EPCs due to their phenotype and angiogenic properties.

### 3.2. Mature Organospecific ECs

#### 3.2.1. HUVECs

Human umbilical vein endothelial cells (HUVECs) have been the most frequently used in vitro models in oncology or cardiovascular EC research since 1973, when they were described for the first time by Jaffe E. et al. [85]. These primary cells are obtained by collagenase digestion of the interior of the human umbilical vein, could only be cultured up to a few months (5 months) because they undergo cellular senescence which finally leads to cell death [85,86]. Due to this fact, only short-term research could be performed on with HUVECs as models. However, Folkman J. et al. proposed a culture up to 8 months for longer experiments, but still different donors of the cell line were sources of errors [87]. This heterogeneity of donors results in complication when the data are compared [88]. It has been shown that responsiveness to IL-8 may vary between culture conditions of HUVECs [88]. Moreover, the vascular origin plays a crucial role in morphology of the cells. Human placental endothelial cells (HPEC) are more elongated and form networks in low density cultures compared to human umbilical vein endothelial cells. Taken altogether, it could be advised to use the immortalized endothelial cell line which will be a model of experiments giving repeatable and comparable results [88].

To avoid all limitations of primary cultures of ECs described above and to investigate large-vessel endothelium, another cell line was obtained—EA.hy926—by the fusion of HUVECs with A549 (human lung carcinoma cell line) [89]. This cell line expressed only one marker of differentiated vascular endothelium, factor VIII-related antigen. Although, Edgell C. J. et al. established EA.hy926 cells that maintained VIII-Ag for 100 cumulative population doublings, including more than 50 passages and three cloning steps, the absolute criteria for predicting the immortality of this cell line were not established [89]. Ahn. K. et al. demonstrated that EA.hy926 preserves the endothelin converting enzyme (ECE) activity of HUVEC and is useful for the study of ECE and its regulation of endothelin-1 (ET-1) production [90]. EA.hy926 has been used as a model in the research investigating ethyl pyruvate (EP) as an inhibitor of LDL oxidation, which is a central element in the development of atherosclerosis [91]. Furthermore, this cell line was used to evaluate the effects of grape seed proanthocyanidin extract (GSPE) under high glucose condition [92]. Taken altogether, EA.hy926 is a permanent human cell line that is widely used in the EC research, though not being well characterized in the criteria of endothelial markers (CD202b, CD146, CD105, CD143), markers of finally differentiated endothelial cells (CD31, VWF, CD45) and activity (pseudo-tube formation, wound healing).

#### 3.2.2. Adult ECs from Various Sources

Primary ECs have been described according to their origin: artery or vein, macrovascular or microvascular vessel. Accordingly, we can distinguish macrovascular cardiac ECs as the following: human aortic endothelial cells (HAEC) [93], human coronary artery endothelial cells (HCAEC) [94], human pulmonary artery endothelial cells (HPAEC) [95]. The microvascular ECs isolated from humans are: human dermal microvasculature endothelial cells (HDMEC) [96], human pulmonary microvasculature endothelial cells (HPMEC) [97], human brain microvasculature endothelial cells (HBMEC) [98,99].

To demonstrate the effective organo-specificity of endothelial cells, tissue-specific ECs were isolated, characterized phenotypically and functionally by Kieda C. et al. [4]. These include ECs isolated from peripheral lymph nodes from surgical biopsies: HPLNEC.B3 (cervical), HPLNEC.S1R1 (inguinal), HMLNEC.MEL (mesenteric); cells isolated from patient with ovarian carcinoma (HOMEC.J6B), appendicitis (HAPEC.S1), cells originated from normal skin (HSKMEC.1) microvasculature and normal intestine (HIMEC.1). Furthermore, immortalized HS888Lu cells from normal lung tissue of a patient with osteosarcoma metastatis to the lung (HLMEC) were described [4]. These cell lines, maintaining their organo-specific characteristics, were well characterized (Patent number: 9916169A, 9631178). All cell lines were positive for ACE, VE-cadherin and E-selectin what confirms that they maintain their endothelial phenotype. P-selectin positive cells, EC activation marker, were found in the following cell lines: HSKMEC.1, HAPEC.S1 and HIMEC.1 [4]. Furthermore, other markers, related to the EC maturation level or organ of origin, were tested: CD34 (positive cell lines: HPLNEC.B3, HPLNEC.S1R1, HOMEC.J6B), CD54 (positive cell lines: HSKMEC.1, HMLNEC.MEL, HAPEC.S1, HIMEC.1), CD49e (positive cell lines: HSKMEC.1i, HAPEC.S1, HIMEC.1) and CD44 for which all cell lines were negative, except HIMEC.1 [4]. These phenotypes were maintained after immortalization what makes described cell lines a valuable tool to study organo-specific ECs in long-term in vitro experiments (Patent Number 9916169A).

Another cell line that omits limitations of primary cultures and donor-dependent variable results used in microvascular endothelium studies, is HMEC-1, obtained by transfection of human dermal microvascular ECs (HDMECs) with SV40T sequences [100,101,102]. Yuelin Xu et al. demonstrated that HMEC-1 constitutively expressed platelet-endothelial cell adhesion molecule (CD31), ICAM-1 (CD54), LFA-3, and MHC class I but lacked of CD36 (thrombospondin receptor), NCAM, and MHC class II. These phenotypic characteristics were identical to those of HUVECs but differed from HDMECs, which expressed both CD36 and NCAM HMEC-1 EN4, PAL-E, H3/5-47, and CD36 [102]. They also express the cell adhesion molecules ICAM-1 and CD44 [100]. Moreover, early studies indicated morphologic similarities with HDMECs and that HMEC-l formed tubes on Matrigel^TM^, took up Ac-LDL, and expressed VWF. These characteristics remained stable, with the exceptions of VWF and CD36. HMEC-1 no longer expresses either of them, although mRNA for VWF is still present [102]. Ades. E. et al. have established HMEC-1 and present that the cell line might be passaged more than 95 times and does not undergo senescence, showing a life span 10-times longer than the primary culture [100,101]. Muñoz-Vega, M. et al. showed that HMEC-1 present similar morphology, size and granularity of the cells as HUVECs. HMEC-1 expressed ICAM-1 and VCAM-1 after TNF-α stimulation [101]. Moreover, Kryczka J. et al., showed that HMEC-1 adopt the mixed amoeboid-mesenchymal migration type during EndoMT (Endothelial-to-Mesenchymal Transition) (downregulation of the endothelial marker VE-cadherin) [103].

The next promising cell lines to study microvascular endothelium are dermal vascular endothelial cells that were immortalized by stably expressing human telomerase catalytic subunit hTERT (hTERT-HDMEC) [104]. All hTERT-EC lines resembled young primary ECs in their morphology and growth response, with little or no staining with SA β-galactosidase activity, defining hTERT-EC line as immortalized [104]. Moreover, data indicate that hTERT(+) ECs retain EC characteristics such as: expression of Von Willebrand factor, high PECAM-1 reactivity, LDL uptake. In the addition, TNF-α-stimulated cell surface expression of ICAM-1, VCAM-1 and E-selectin [104]. Data also show pro-angiogenic responses on 3D (three-dimensional) collagen culture by formation of tubes in vitro [104].

Besides the human cells, similar endothelial cells’ immortalization and lines establishment in conditions that keep the phenotype stability were performed from murine, canine and bovine origins, which not only allowed the confirmation of the high degree of organo-specificity, but also demonstration of the species-specificity of infectious diseases [105]. Moreover, the immortalization of cell lines was performed from cat and eleven lines were obtained from distinct tissue origin (Patent number: 9228173). The feline skin endothelial cells (FSkMEC) derived from the microvasculature and the feline umbilical cord endothelial cells (FUmEC) derived from the macrovasculature were used to demonstrate the cellular process of specific EC skin bacterial invasion by Bartonella Hansellae in cats and the species specific transmission to human [105]. The similar cell line establishment strategies in bovine were performed to study, in proper models, the organ and specific molecular mechanisms of viral infections [106].

Considering the above described characteristics of cells, the use of HUVECs as a standard model is risky because it does not reflect the in vivo state and ECs’ heterogeneity. Furthermore, the isolation of organospecific cells is a new way to obtain cell models for in vitro (iPSCs- induced pluripotent stem cells) that are more relevant to in vivo conditions. It opens to future use in personalized medicine as a tool for targeted therapy.

### 3.3. iPSCs Derived Endothelial Cells

The possibility of reprogramming somatic cells into pluripotent stem cells by introducing the genes coding for four factors, Oct3/4, Sox2, c-Myc and Klf4, described by Yamanaka S. and Takahashi K. [107,108] did facilitate advances in regenerative medicine, but also contributed to the development of new strategies in in vitro research. iPSCs (induced pluripotent stem cells) have the potential to differentiate into other cells including endothelial cells, which allows for generating patient-specific ECs and their uses in in vitro tests as well as taking advantage of their therapeutic potential. Many methods of iPSC differentiation into endothelial cells have been developed, including: co-culture with stromal cells, embryoid body (EB), two-dimensional culture on a matrix-coated surface with the addition of appropriate molecules and growth factors as well as three-dimensional culture [109,110]. Choi K.D. et al. co-cultured various human iPSC cell lines with OP9 feeders for 8 days, which resulted in the generation of hematopoietic precursors and endothelial cells (CD31+, CD43−). These ECs also expressed CD105, CD144, VE-cadherin, VEGFR2, and were also able to form tubes on Matrigel^TM^ [111]. Similar results were obtained by Taura et al., but in both cases a low efficiency (<6%) of differentiation was noted and the resulting cell culture was strongly heterogeneous [112]. Better efficiency was obtained using the embryoid body (EB) differentiation relying on the spontaneous differentiation of aggregated iPSC. Adams W.J. et al. characterized the timescale of EC differentiation from EBs—after 10 days they obtained 18 ± 4% CD144 and CD31 positive cells which are typical mature EC makers. In addition to assessing endothelial-specific markers (presence of CD144, CD31, VEGFR2 and others), the authors also evaluated the functionality of such differentiated ECs. Cells were able to form pseudo-vessels on Matrigel^TM^; in response to inflammation factors the expression of adhesion molecules (E-selectin, ICAM-1, VCAM-1) was upregulated, which is critical for endothelial cells-to-leukocyte interactions and they also released several proinflammatory cytokines and chemokines. Cells were able to interact with T lymphocytes and neutrophils. iPSC-EC barrier capabilities were reduced after the addition of histamine and VEGF, and increased after PGE2 stimulation [113]. Rufaihah A.J. et al. showed that ECs obtained using the EB differentiation method could be directed for differentiation into: (a) arterial iPSC-ECs, using higher concentrations of VEGF-A and 8-bromoadenosine3′:5′-cyclic monophosphate; (b) venous phenotype with low concentrations of VEGF-A; and (c) lymphatic, using VEGF-C and Ang1 (angiopoietin-1) [114]. Arterial and venous endothelial cells were also obtained under biochemically defined conditions, in monoculture or seeded in a scaffold by two-stage differentiation—first into mesoderm precursors, and then into endothelial precursor cells (EPCs), which express residual venous and arterial markers [115]. Despite the apparent possibility to modulate the differentiation of one differentiated cell into another, Hu S. et al. showed that the differentiation of ECs depends on the origin of iPSC—from one patient they obtained three different iPSC lines (from fibroblasts (FB-iPSC), endothelial cells (EC-iPSC) and cardiac progenitor cells (CPCiPSC) and differentiated them into ECs using activin bone marrow protein 4 (BMP4), basic fibroblast growth factor (bFGF) and VEGF. Among the three lineages, EC-iPSC had the highest ability to differentiate into ECs. This was further confirmed functionally showing the importance of the cellular origin which dictates the lineage differentiation propensity of iPSCs [116]. This demonstrates the limit of iPSC potential, the importance of tissue specificity as well as the limit of the ability of differentiation. Called the somatic memory of iPSCs, this takes its importance and must be taken into account to delineate the application to clinical translation.

Although the effect of miRNA on endothelial cells has already been discussed, there is little evidence on the role of these small, non-coding RNAs in iPSC-EC. MicroRNA-21 mediates endothelial differentiation from iPSCs in the presence of VEGF—overexpression of miR-21 in iPSCs induced EC marker up-regulation accordingly, inhibition of miR-21 produced the opposite effects. The direct target of miR-21 is the PTEN/AKT pathway and *PTEN* knockdown is required for endothelial differentiation via miR-21 [117]. miR199a also affects EC differentiation—the use of a mimic of this miRNA increased expression of CD144 and CD31 as well as the ability to form tubes on Matrigel^TM^ [118]. Wang L. et al. identified several miRNAs: miR-125a-5p, miR-149, miR-296-5p, miR-100, miR-27b, miR-181a and miR-137, which were up-regulated in hiPSC-ECs during endothelial differentiation [119]. Numerous studies have compared the functional potential of iPS-derived ECs with the standard HUVECs used; however, the results are not conclusive. Among others, it has been shown that iPSC-ECs displayed a five-fold reduction in capillary network formation compared to HUVECs when co-cultured with human lung fibroblasts NHLFs in a 3D fibrin matrix, at the same time it has been proven that it is associated with a weaker expression and activity of MMP-9 (matrix metallopeptidase 9) [120]. Other studies on hiPSC-EC functionality in the vasculogenesis by in vivo testing, showed that CD34 + hiPSC and CD31 + hiPSC formed very dense sprouting networks with numbers of junctions and total vessel lengths significantly higher than HDMEC and HUVEC [121]. Moreover, iPSC-derived endothelial cells displayed in vitro inflammatory responses comparable to primary cells—an increase of ICAM-1 and E-selectin levels after stimulation with TNFα was observed, although the leukocyte adhesion ability was lower than towards HUVECs [121]. In the in vivo model of zebrafish, iPSCs displayed a greater ability to integrate with the vascular system than HUVEC [122].

Obtaining organospecific endothelial cells from iPSC seems to be a very promising solution, especially for tissues from which it is difficult to isolate primary ECs. To date, several protocols obtaining brain-types of microvessels from iPSC have been developed. These cells display many attributes that permit to reconstitute in vitro a blood–brain barrier model: high expression of adherent and tight junction proteins, VE-cadherin, ZO-1, Occludin and Claudin-5, as well as transporters (LAT-1) and efflux pumps (P-glycoprotein). Such a model also presents appropriate permeability indicators: TEER values after stimulation with retinoic acid or by co-culture with astrocytes do reach maximal levels above 1000. These are comparable to levels reported for rat brains in vivo (1000–1500 Ω cm^2^) [123,124,125]. Patient-specific brain endothelial cells obtained from iPSCs are a promising model for studying the underlying mechanisms and potential therapy of neurological disorders. Lim R.G. et al. showed that in Huntington’s disease, endothelial cells have intrinsic impairments in angiogenic potential and drug efflux capacity, they form abnormal blood–brain barrier, and have WNT signaling defects [126].

This method of differentiating iPSC also allowed for the production of other organospecific types of ECs, among others cardiovasculature-specific endothelial cells (expressing markers MEOX2, GATA4, GATA6 and ISL1) [127], as well as corneal endothelial cells [128] or coronal-like ECs [129]. However, the lineage somatic memory and the primary cells instability in culture should be taken into account in the iPSC dedifferentiation assays. This was particularly demonstrated in the EC line production and gets more significant considering the endothelial cells organospecificity [4]. As described earlier, EC specific phenotype and function in distinct organs are key to proper biological phenomena and homeostasis. This demonstrates the importance of careful consideration of the biological environment of a cell in the organ it is located in, together with its plasticity as a function of its origin.

### 3.4. The Limitations of Primary Isolated Cells and Cell Lines

In many cases, immortalized ECs offer a very valuable model for preliminary in vitro studies to omit disadvantages of primary cell lines. These include the presence of contaminating cells and limited numbers of cells, as well as the progressive loss of cell viability [130]. On the other hand, many cell lines with extended life span showed disadvantages: tumorigenicity or chromosomal instability, loss of primary endothelial features [131]. Checked for non tumorigenicity, transformed cells with a longer life span may still present different transcriptomes and phenotypes from the tissue of origin when compared to primary ECs which save important markers and functions more relevant to human physiology [132]. Although primary ECs present such advantages, they also undergo fast aging processes [133]. Thus, they show morphological and functional changes and have limited potential for self-renewal and differentiation and are known to lose their specific characters over 4 to 5 passages. Nevertheless, the choice of a suitable model should be chosen according to the project assumptions.

## 4. Endothelial Cells in Advanced In Vitro Models

The widespread presence of endothelial cells in tissues and organs and the importance of the vascular system in the pathophysiology of many diseases resulted in the need to reconstitute in vitro models based on ECs, taking into account their characteristic, organo-specific and microenvironment-dependent plasticity in: co-cultures, invasion/migration assays, angiogenesis, 3D (three dimensional) culture, organoids, organ-on-chips. These methods allow better imitation of in vivo conditions [72]. They intend to consider the presence and the role of the various relevant types of cells and their crosstalk, together with the extracellular matrix components, oxygen partial pressure conditions and variations or shear stress-fluid flow. They provide a good alternative to study the mechanisms of diseases and the design of new therapies.

Advanced methods are used to study, among others, the mechanisms of tumor angiogenesis. Buchanan C.F. et al. showed that co-culture of breast cancer cells and endothelial cells (telomerase-immortalized human microvascular endothelial cell line—TIME) in 3D microfluidic collagen hydrogel increases the permeability of ECs-formed structures. Changes in gene expression associated with angiogenesis (VEGF, MMP, ANG1, ANG2) depend on the values of microvascular wall shear stress, resulting, among others, in changes in ECs’ permeability [54]. In the 3D models of melanoma cells, Klimkiewicz K. et al. showed the recruitment of both mature and progenitor endothelial cells to tumor spheroids (in both mouse and human models) [72]. This recruitment was an active process, observed only for live cancer cells. ECs’ recruitment by melanoma spheroids were higher in hypoxia than in normoxia indicating the hypoxia-dependent signaling specific for early endothelial progenitors (MAgEC 10.5). Moreover, in normoxia the recruitment process of ECs began with a delay, and the previously non-organized ECs evolved in the long term into a complex network suggesting the appearance of hypoxia inside the spheroid, which also shows the sensitivity of endothelial cells to the aerobic conditions of the micro environment [72]. The 3D model with endothelial cells (HUVEC) was also developed for hepatocellular carcinoma and used to study the effectiveness of anti-angiogenic drugs (sorafenib, sunitinib, axitinib) [134]. Although the authors have proven that the 3D model well reflects the hypoxic core of the tumor, true gradient penetration of drugs and observed that all three drugs prevented the formation of vessels, it is necessary to mention the limitations: vessels remain stable in this model only for a short time (3 days) due to, among others, lack of pericytes, fibroblasts and vascular smooth muscle cells. This makes the researchers unable to investigate the long-term effect of anti-angiogenic drugs. This could be a key effect as Pàez-Ribes M. et al. indicate that the effect of anti-angiogenic drugs is temporary, later on the tumor becomes resistant to such treatment, what leads to revascularization, induction of more aggressive phenotype and increases metastasis [135].

Another use of endothelial cells in 3D models and co-culture with other cells is to create an in vitro blood–brain barrier (BBB). Wilhelm I. et al. established a rat in vitro BBB model using Transwell filters with primary rat brain endothelial cells (RBECs), pericytes and astrocytes. After stimulation with hydroxycortisone cAMP (cyclic adenosine monophosphate) and RO (4-(3-Butoxy-4-methoxybenzyl) imidazolidin-2-one)) they obtained high TEER values of 264 ± 67 Ohm × cm^2^ and low permeability, which makes this model useful for drug transport testing [136]. Stone N.Z. et al. point to the need to enrich the three-cell model with neurons which make BBB integrity more sensitive to oxygen-glucose deficiency, as evidenced by a decrease in TEER, increased permeability and markers of cell damage [137]. Another method of recreating the BBB in in vitro conditions using ECs in co-culture are self-assembling spheroids, of which the core is comprised mainly of astrocytes, while brain endothelial cells and pericytes encase the surface, acting as a barrier that regulates transport of molecules. The spheroid surface exhibits high expression of tight junction proteins ZO-1, Claudin5, Occludin, VEGF-dependent permeability, efflux pump activity and receptor-mediated transcytosis of angiopep-2 [138]. The more advanced 3D microfluid BBB model was designed by Campisi M. et al. based on the ability of hiPSC derived ECs to differentiate and self-assemble into brain microvascular cells in cooperation with pericytes and astrocytes which, by facilitating the organization of the endothelium, stabilize the mature vascular system, with tight connections and low permeability [139].

## 5. Endothelial Cells in Angiogenesis and the Role of ECs in Pathologies

The key players of angiogenesis are endothelial cells (ECs) which form blood vessels with other cell types, called mural cells, that include the vascular smooth muscle cells (vSMCs) and pericytes [140]. Endothelial cells are responsible for the regulation of transport across the vessel [141]. The pathological angiogenesis, one of the hallmarks of cancer plays a crucial role during solid tumor growth and metastatic spread [142]. Angiogenesis is stimulated by chemical signals from tumor cells in a phase of rapid growth when nutrients and oxygen must be provided. The imbalance between inhibitors (among others: angiostatin, endostatin, interferon) and activators of this process is caused by several factors including: hypoxia, low pH, hypoglycemia, immune/inflammatory stimuli [143]. These signals come from the angiogenic activators, including: growth factors (VEGF-A, bFGF), interleukin-8, angiogenin, transforming growth factor (TGF)-α, TGF-β, tumor necrosis factor (TNF)-α, platelet-derived endothelial growth factor, granulocyte colony-stimulating factor, placental growth factor, hepatocyte and epidermal growth factor [143,144]. Among the variety of these angiogenic modulators, VEGF-A is the essential player in the formation of blood vessels.

In low pO_2_, hypoxia present in the growing tumor mass has the direct effect to increase expression of hypoxia-inducible factor 1 (HIF1α) induces VEGF-A synthesis triggering ECs’ activation by binding to VEGF receptors on the ECs’ surface [145]. Tumor cells release VEGF-A which stimulates ECs to break down the basement membrane in blood vessel by the release of proteolytic enzymes [146]. Plasmin, which is the active form of plasminogen, activated by urokinase-plasminogen activator (uPA), is one such key proteolytic enzyme [147]. The activation of endothelial cells from preexisting blood vessels to form new vessels in hierarchical manner is mediated by motile endothelial cells located at the ends of newly formed vessels, known as tip cells [148,149]. The other endothelial cells that follow the migrating tip cell, differentiate under the influence of the tip cell into stalk cells that proliferate and create a lumen [148]. When, endothelial cells adhere to one another and lumenogenesis takes place, the basement-membrane formation and pericyte attachment occurs.

**Figure 3:**
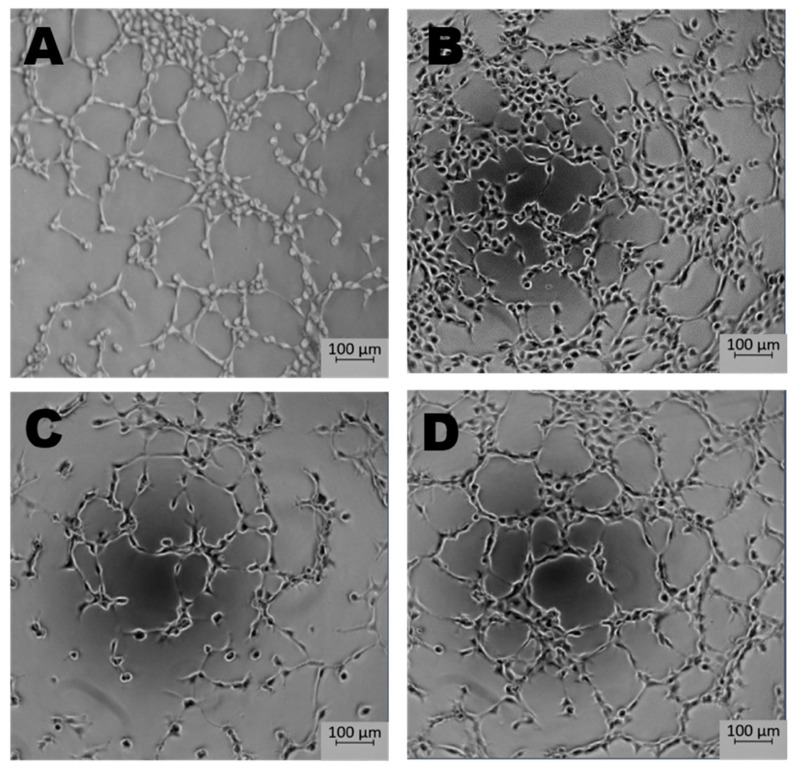
The effect of microenvironment and organospecificity on the angiogenic potential of Endothelial cells (ECs). Morphology of tubes formed in vitro by healthy and cancer-derived EC cells after 5 h in normoxia or hypoxia. Low pO_2_ reduces the pro-angiogenic response in ECs derived from tumor site when compared to normoxia. In the case of healthy tissue-derived ECs, hypoxia does not significantly influence tube formation. (**A**)—healthy ECs, normoxia; (**B**)—healthy ECs, hypoxia; (**C**)—cancer-derived ECs, normoxia; (**D**)—cancer-derived ECs, hypoxia; magnification 5×.

**Figure 4:**
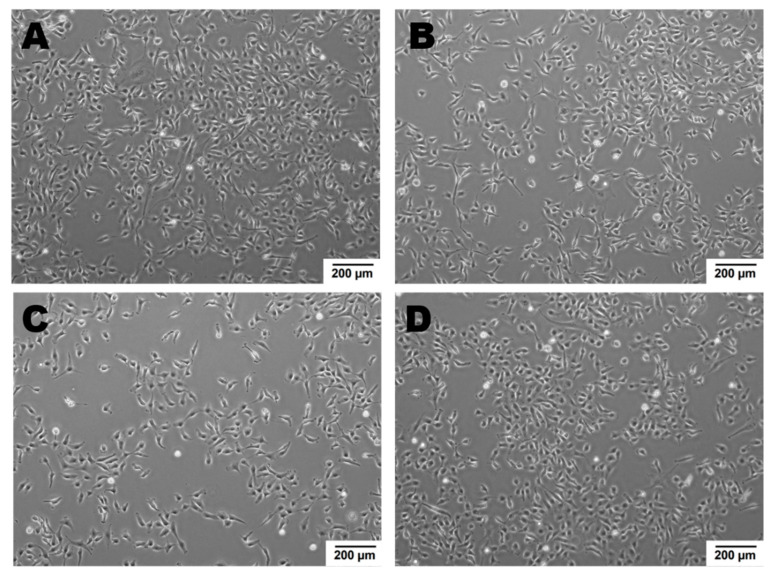
ECs’ growth modulated by hypoxic micro-environmental conditions. Cell density after 48 h in normoxia or hypoxia: Cells were seeded at 0 h in the same number. Growth of healthy ECs is a little slower upon hypoxia vs. normoxia, oppositely to cancer derived ECs, whose growth is higher in hypoxic conditions as compared to normoxia. (**A**)—healthy ECs, normoxia; (**B**)—healthy ECs, hypoxia; (**C**)—cancer-derived ECs, normoxia; (**D**)—cancer-derived ECs, hypoxia; magnification 4×.

Newly formed blood vessels should supply nutrients and oxygen for tissue; however, in the tumor, their permeability is disrupted due to pathologic structure and differences between ECs originated from healthy and pathological site [150]. Firstly, the cells are structurally organized in different ways. Although, ECs are structurally heterogenous and functionally dependent on their origin called organospecificity, in general healthy ECs form layers sealed by tight junctions on the luminal surface of the vessel [4,150]. On the contrary, cancer-derived ECs generate chaotic formation of the inner cellular lining of blood vessels [151]. Moreover, the pathologic endothelium is immature and thin-walled [152]. Secondly, the permeability and perfusion vary between healthy and pathologic vessels [153]. The way the vessels are structured defines their function. In healthy-derived endothelium, the ordered layers allow easy flow and free passage of blood. Oppositely, pathological EC structure is associated with increased permeability and decreased perfusion in the blood vessel [154]. Thirdly, tumor endothelial cells have even higher glycolytic metabolism comparing to the healthy ones [33,155]. Last but not least, the differences between tumor suppressors and oncogenes might be the reason of the dissimilarity described above. It has been shown that in pathologic ECs, the main controller of angiogenesis—PTEN, tumor suppressor gene (endothelial phosphatase and tensin homolog) is one of the most deregulated genes [156,157]. Moreover, in pathologic angiogenesis, VEGF synthesis is increased and permanently activated by hypoxia at the tumor cells level. This maintains the angiogenic anarchic growth and results in pro-tumorigenic signals [156,158,159]. Such disrupted phenotype of pathological vessel can be maintained in vitro. Indeed, ECs isolated from a tumor site differ from healthy ones on molecular and functional level (Figure 3, data not published). Additionally, in vitro hypoxia (culture of cells in low, non-physiological pO_2_, usually 1%) is an appropriate tool to mimic tumor micro-environmental conditions [160,161,162]. Figure 4 displays the observed growth differences evidenced upon hypoxia treatment of the cells, while cells differences appear in their ability to form vessels in vitro estimated by functional assay for pseudo-tube formation (data not published, Figure 3). Understanding the mechanisms related to pathological angiogenesis in the tumor microenvironment revealed ECs as a new, potential therapeutic target for the treatment of many solid tumors. In 2004 the US Food and Drug Administration approved the first VEGFA inhibitor, bevacizumab for the first-line treatment of metastatic colorectal cancer [163]. The new developments in this area contributed to the identification and approval of many anti-angiogenic drugs, which offered better outcomes (often in combination with standard chemotherapy or immune checkpoint blockers) [164,165]. The aim of anti-angiogenic therapies is mainly to inhibit VEGF-VEGFR signaling at different levels: (a) blocking the activity of VEGFR by tyrosine kinase inhibitors (TKIs) (b) neauralizing circulating VEGF molecules with a monoclonal antibody (c) blocking the activity of VEGFR by monoclonal antibody [166,167,168]. However, the effectiveness of anti-angiogenic therapies in the light of recent reports and clinical observation is being more and more often denied [166,169]. Effects of anti-angiogenic treatment in vivo is only temporary which is mediated by hypoxia-resistance mechanisms such as: inducing new vascular mimicry properties by tumor cells, increased production of proangiogenic factors, or autophagy [170,171,172]. Studies show that such therapy can in fact, promote the metastatic potential of tumor by increasing collagen deposition [173], modification of its properties and show harmful clinical side effects (ex. Bevacizumab—a monoclonal antibody that neutralizes the vascular endothelial growth factor A (VEGF-A) [174,175]. RTKIs, which inhibit the vascular endothelial growth factor receptors (VEGFRs) tend to be insufficient [168]. Due to this fact, it is extremely important to develop therapies that will be more effective and durable. One of the approaches is to alleviate hypoxia by bringing back proper permeability and perfusion of the vessels. Such normalization of the vessels might be an invaluable tool to improve the outcome of anti-cancer therapies by reconstituting the vessels proper function, thus increasing chemotherapeutics penetration [176,177]. Another potential tool in cancer therapy is offered by microRNAs-mediated (angiomiRs) regulation that modulate angiogenesis, regulate cancer immunity (miR-424) and can be combined with T cell-based therapy (miR-17-92,-155,-181a) or suppression of VEGF (miR-16-like family) and interference with TGF-β signaling [47,162,178,179,180]. MicroRNA-34a has been reported as an anti-metastatic and suppresses angiogenesis in bladder cancer by directly targeting CD44 [181]. An interesting concept is also the attempt to use precursor endothelial cells to deliver therapeutic miRNAs to the tumor site [182].

Based on the need for an efficient vessel normalization, strategies are developed to increase oxygen partial pressure in the tumor site. It has been shown that allosteric effector of hemoglobin (Hb), *myo*-inositol trispyrophosphate (ITPP) inhibits hypoxia-induced pathological angiogenesis by activation of PTEN in the ECs [156,183]. ITPP primarily acts as an allosteric effector enhancing the capacity of hemoglobin to release bound oxygen, which influences vessel normalization by reducing the HIF-1α activation thus reducing the VEGF-A synthesis [183,184]. The regulation by PTEN, was shown. As PTEN is the main control of the PI3K/mTOR/p53 axis, ITPP based strategies can be a key method for angiogenesis regulation and vessel normalization. Additionally, PTEN activation which leads to the inhibition of PI3K (phosphoinositide 3-kinases) and decreasing tumor AKT (protein kinase B)phosphorylation results in tumor growth reduction [156]. Moreover, ITPP was described as an effective tool in the treatment of hypoxia-dependent cardiovascular diseases (heart failure) by similar molecular mechanisms [185,186].

Another blood vessel normalization strategy, resulting from tumor hypoxia alleviation, is based on cell-gene therapy with the use of endothelial progenitors in murine model (MAgEC11.5) [69]. These progenitors are able to target pathological angiogenesis and by cell mediated gene therapy allow the expression, locally, of soluble VEGF-receptor2 in a hypoxia-driven manner. It acts as a VEGF trap and its action results in vessel normalization and functionalization, microenvironment composition modification and ultimately, in tumor growth reduction [69]. This is a direct demonstration and application of angiogenesis normalization cell-gene therapy.

In addition, Alk1 (activin receptor-like kinase 1) has been described as another therapeutic target which is responsible for promoting vascular remodeling and maturation [187]. It has been proven that the Alk1 ligand -BMP9- promotes vascular normalization in Lewis Lung Carcinoma (LLC) tumors what results in deep microenvironment changes [187].

Moreover, the combination of immunotherapy with anti-angiogenic treatment in cancer has been described. Recently, most clinical trials have been devoted to PD1 (Programmed cell death protein 1)/PD-L1 (Programmed death-ligand 1) immune checkpoint neutralization. PD-L1 is largely used as the anti-tumor immunity target and VEGF-A is the anti-angiogenic target which are applied in trials concerning non-small cells lung carcinoma (NSCLC), renal cell carcinoma ovarian cancer patients and many other cancers as melanoma [165] where the data, although promising, remain partial.

This points to the deep interest that should be devoted to conducting experiments and alternative assays in conditions as close as possible to those encountered in vivo, namely hypoxia, which appears fundamental for angiogenesis-based studies.

## 6. Conclusions

To sum up, the choice of proper in vitro cellular models to study pathologies by alternative methods is crucial to perform biologically relevant research involving endothelial cells. Several in vitro tools are accessible to properly mimic ECs’ heterogeneity, organo-specificity and plasticity in response to environmental stimuli. Growth and unification of methods of isolation and culture of ECs may help elucidate the mechanisms of endothelium development and function in health and disease, which in turn, can bring effective adaptations for the development of new treatment strategies for angiogenesis-dependent diseases. The large and fast increase in the endothelial cell lines devoted to defined studies, points to the great advantage of using comparable sets of similarly treated cells. The main routes opened by the knowledge of the endothelial cells organo-specificity is the development of cell-carried tissue-specific therapies for repair strategies. As alternative methods are being developed, competition favors the models that present properties as close as possible to the in vivo context in terms of micro-environmental parameters as well as dynamics effects. The importance of hypoxia in diseases is getting primordial, and pathologies classified as hypoxia-dependent are increasing. This cannot be dissociated from angiogenesis as the most direct effect of hypoxia and for its consequences when abnormal. Means for the reconstitution of proper vessels are essential to learn about the potential and effects of a successful repair of the pathologic vasculature. In this line, advantages provided by the early endothelial progenitor cells lie in their ability to target the hypoxic sites. They naturally counteract and compensate the damaged cell wall and may reconstitute homeostasis. In addition to such cellular processes, this review points to the molecular modifications of the pathologic angiogenesis and shows how this may help to, consequently, succeed in the control of the tumor microenvironment through hypoxia compensation.

## Figures and Tables

**Table 1:** Cellular markers for the phenotyping of endothelial cells.

Marker	Characteristic	Reference
CD 31 (PECAM-1)	Platelet endothelial cell adhesion molecule that localizes the endothelial cell intercellular junction, is involved in the migration of leukocytes, and plays a role in angiogenesis	[8,16]
VEGFR2 (CD309, FLk-1, KDF)	Vascular endothelial growth factor receptor 2 transmembrane receptor tyrosine kinase that triggers angiogenesis; networks initiated by VEGF-A/VEGFR2 leads to endothelial cell proliferation, migration, survival and new vessel formation involved in angiogenesis	[17]
VEGFR3	Vascular endothelial growth factor receptor 3 transmembrane receptor tyrosine kinase; characteristic marker of lymphatic endothelial cells; VEGFR3 and its ligands (VEGF-C and VEGF-D) are involved in lymphangiogenesis and by forming complexes with VEGFR2 plays a role in angiogenesis	[18]
CD144 (VE-cadherin)	Endothelial specific adhesion molecule responsible for junction between cells, inhibition of VE-cadherin increases monolayer permeability and enhances neutrophil transendothelial migration	[9]
VWF	Von Willebrand factor (VWF) is a glycoprotein released from Weibel–Palade bodies (WPB) of endothelial cells and is associated with blood clotting by stabilizing factor VIII	[19]
EphB4	Receptor tyrosine kinase, marker of adult venous ECs	[13]
Ephrin-B2	Transmembrane ligand for EphB4, marker of arterial endothelial cells	[13]
CD 54 (ICAM-1)	Intercellular adhesion molecule-1 is involved in adhesion of immune cells during inflammation	[16]
CD106 (VCAM-1)	Vascular cell adhesion molecule-1 is involved in adhesion of immune cells during inflammation	[16]
CD146 (MCAM)	Melanoma adhesion molecule facilitates cell-cell interaction and is involved in inflammation and angiogenesis	[20]
CD105 (Endoglin)	Receptor for transforming grow factor β (TGF-β) affects angiogenesis by regulating ECs proliferation; induces the anti-apoptotic pathway of ECs in hypoxia	[21]
CD62e (E-selectin)	Endothelial leukocyte adhesion molecule-1, glycoprotein from the family of selectin (E-selectin, L-selectin, and P-selectin), it is expressed in endothelial cells after stimulation by TNF-α (tumor necrosis factor alpha), Il-1 (interleukin 1) or bacterial lipopolysaccharides, main player in early and specific adhesion of immune cells	[16]
Podoplanin	Membrane glycoprotein of podocytes, specific marker for lymphatic endothelial cells, plays a role in the regulation of lymphatic vascular formation and movement	[22]
LYVE-1	Membrane glycoprotein capable of binding to hyaluronic acid, marker of lymphatic endothelial cells	[12]
CD44	Cell surface adhesion receptor, is a marker of late endothelial progenitor (EPC) cells plays a role in ECs’ regeneration	[23]
CD34	Glycoprotein first identified on hematopoietic stem and progenitor cells but it is also present in most micro-vessels in the umbilical artery but not in the endothelium of large vessels	[24]
CD133 (Prominin-1)	Tissue-specific stem cell marker, characteristic for EPCs	[10]
CD202b (Tie-2)	Hematopoietic stem cells marker also present in EPCs, receptor for Ang-1 and Ang-2	[25]

## Abbreviations

2D	two-dimensional
3D	three-dimensional
Ac-LDL	acetylated low-density lipoprotein
ACE	angiotensin converting enzyme
ALK1	activin receptor-like kinase 1
Ang	angiopoetin
BBB	blood-brain barrier
bFGF	basic fibroblast growth factor
BMP	bone morphogenetic protein
cAMP	cyclic adenosine monophosphate
CCL21	chemokine (C-C motif) ligand 21
CX3CL1	fractalkine; chemokine (C-X3-C motif) ligand 1
CXCR4	chemokine receptor type 4
EB	embryoid body
EC	endothelial cell
EndoMT	Endothelial-to-Mesenchymal Transition
eNOS	endothelial nitric oxide synthase
EP	ethyl pyruvate
EPC	endothelial progenitor cell
EphB4	ephrin type-B receptor 4 gene
ET-1	endothelin 1
GSPE	grape seed proanthocyanidin extract
HIF1	hypoxia-inducible factor 1
HUCB	human umbilical cord blood
ICAM-1	intercellular adhesion molecule-1
ILs	interleukins
iPSC	induced pluripotent stem cell
ITPP	myo-inositol trispyrophosphate
LFA-3	lymphocyte function-associated antigen 3
LLC	Lewis Lung Carcinoma
LYVE-1	lymphatic vessel endothelial hyaluronan receptor-1
MCAM	melanoma cell adhesion molecule
MHC	major histocompatibility complex
miRNAs	microRNAs
MMP-9	matrix metallopeptidase 9
MNC	peripheral blood mononuclear cell
NCAM	neural cell adhesion molecule
NSCLC	non-small cells lung carcinoma
PECAM -1	platelet endothelial cell adhesion molecule
PGE2	prostaglandin E2
PI3K	phosphoinositide 3-kinases
PODXL	podocalyxin-like protein 1
PTEN	phosphatase and tensin homolog deleted on chromosome ten
RO	4-(3-Butoxy-4-methoxybenzyl) imidazolidin-2-one)
RTKIs	receptor tyrosine kinase inhibitors
Sca-1	stem cells antigen-1
TEER	transepithelial/transendothelial electrical resistance
TNF	tumor necrosis factor
UEA	ulex europaeus agglutining
VCAM-1	vascular cell adhesion molecule-1
VE-Cadherin	vascular endothelial cadherin
VEGF	vascular endothelial growth factor
VWF	von Willebrand factor
WPB	weibel–Palade bodies
ZO-1	zonula occludens-1
